# In the Children's Ward

**Published:** 1887-03-05

**Authors:** Walter J. Eccott


### In the Children's Ward.

By Walter J. Eccott.

Chapter I.

Apartments at 29, Upper Collingwood Street, N.W., were by no means uncomfortable. At least they were not so when George Inman and his wife occupied them. They consisted of two rooms on the first floor, and a small cellar kitchen in the basement. The furniture was not very bright, nor had the pieces much the appearance of having been bought *en suite*. On the contrary, a careful observer would see that they had been bought separately at second-hand, and introduced to one another. But all were substantial and good. And not lightly did George Inman and his wife value their furniture because it had been thus gathered piece by piece in exchange for hard-earned, thriftily-kept money. What a pleasure had it been for Tilda, both in their courting days and since, to go round with George to buy it, after having previously carefully inspected it in some dealer's warerooms!

How the appearance of a room unconsciously reflects the tastes and occupations of its inhabitants! I mean a "living" room, and not one of those useless apartments whose wall-paper is white and cold-looking, which rejoices in a rep-covered walnut (stained) suite, and a round veneered table laid out with books in fancy bindings, where not an article bears traces of having any human associations belonging to it, and where the chance visitor by instinct sits just on the edge of a chair, and departs at the very earliest opportunity. The Inmans' snug little room, at once sitting, dining, and drawing-room, indicated by the number and variety of prettily carved, well-gilt picture-frames and brackets, that George was connected with the practical application of gold-leaf. In fact, he was in the gilder's shop at a well-known decorator's—Cartland, of Cavenmore Street, W. A further glance round the room, and the eye would light on some bookshelves ornamented with some neat gilt mouldings, which bookshelves were not laden with the usual *History of the War* in ten volumes, illustrated, an Encyclopædia in eight volumes, and other books, which are more indicative of the pertinacity of the book-tout than the taste of the possessor. The books were not numerous; they were useful. Two or three works relating to the technique and history of George's craft, a Tennyson, a Shakespeare, some volumes of Dickens and Thackeray, Green's *History of the English People*, Carlyle's *French Revolution*. Any political canvasser wishing to gauge George's politics would have noticed two works issued by "The Cobden Club," Mill's *Political Economy*, and Henry George's *Progress and Poverty*. From these, and the evidences offered by some odd newspapers to be found in a corner, Mr. Inman would have been set down probably as a Radical and a Socialist. He would have pleaded guilty on both counts, and far from attempting to extenuate the fact, would even attempt to convert his accuser. He would tell you he was so by self-education and the force of the times.

It was one of George's plaints that he couldn't drive any notion of politics into his wife, or out of her a great respect for aristocracy. She was a bright, bustling woman of about twenty-eight, who still retained something of the comely roundness of feature and fresh bloom that she wore some four years ago, when George fetched her out of the country. Till he married her she had enjoyed the fresh air and vigorous life on the Hagleycombe estate in Laneshire, where her father was head-gamekeeper. She had exchanged them for somewhat cramped quarters, with not so much as a square yard of garden, and only such air as filtered through the smoke-wreaths that more or less always hung above Upper Collingwood Street. But she had George, and, if he was not compensation enough, she had her one little girl, close upon three years old, at once the anxiety and the delight of both. Proud was George to take Elsie out, dressed in her brightest attire, on a Sunday morning for a walk in Hyde Park, while his wife was preparing the principal meal of the week, to wit, Sunday's dinner.

Mrs. Inman was getting tea ready. She expected her husband every minute. She heard his step on the stair. He came in. From his face she could see something had gone awry with him, but at present she asked no questions. She only noticed that he had brought with him his little bag of tools, which he usually kept in a locker at the "shop." He certainly looked gloomy, but he only opened his lips to ask, rather petulantly, "Where's Elsie?"

"Oh! she's only gone out with Mrs. Millwood's two little girls," said Mrs. Inman. "I wanted to finish my ironing; and they're very fond of her."

"H'm. They're only youngsters. I'm afraid she'll come to some hurt one of these fine days, what with cabs, and people, and telegraph wires."

"Well, I told them not to go beyond Bryanston Square," Tilda said in defence. "Now sit down; tea's quite ready."

George sat down, as desired, and began to eat and drink fast, as a man does when there is something to disturb his mind. But he said nothing.

Mrs. Inman was curious to know what troubled him, but she was quite willing to let him make a good meal before she catechised him.

At length, when he had turned away from the table and sat down in his arm-chair, she began:

"Now, George, what's put you out? Anything amiss at the shop?"

"Yes!" George growled out, "wrong enough! I'm out of work."

"Out of work?" murmured Mrs. Inman, hesitatingly and in a tone of inquiry.

"Yes, out of work," repeated George.

Mrs. Inman said nothing out of pure astonishment. She had regarded George's situation at Cartland's as safe as a Government clerkship, or a gamekeeper's place at Hagleycombe. George had been at Cartland's as long as she had known him.

George went on: "Yes; you see, trade's awful slack just now. Scarcely a job in the shop, and Fred Cartland's been round all the shops to try and make a reduction in the hands. You know old Draycott, our foreman, is no particular friend of mine. He's always been ready to do me a bad turn, especially since he turned Conservative just to please the guv'nor. Old Draycott hates my Radicalism and my arguments, and he's as jealous of my work as he can be. So, when Mr. Fred came to him, on the quiet of course, I'm made out a Nihilist, or at least, as bad as one of those rioters that smashed the shops in South Audley Street—you remember—and one who sets the men against the masters, labour against capital. Of course he told him I worked hard for the Radicals last election, and that, in his opinion, I was a fellow of more talk than work. Mr. Fred doesn't know any better, as he sees very little of our part of the work, and, of course, I have to go. And just when Gillow's, and Crace's, and all the other big firms are turning off hands."

"What a shameful thing!" said Mrs. Inman, with indignation. "Perfect downright wickedness of that Draycott, I declare. And Mr. Fred, who ought to have known better! But what are we going to do?" she went on, in a tone of much concern for the little household. "Do you think you'll get anything to do in a day or two? If you don't get work, how are we going to pay the rent?" Many other questions suggested themselves to her mind, but she only framed these three into words. There is nothing a woman dreads more than bankruptcy, or being behind-hand in her domestic payments.

"Oh! as to that," said George, "for a week or two, or perhaps a month or two, we'll manage to get along without running up scores on tick. You know I've got some money in the bank. But it's bad economy to live on capital."

George was in a better humour now he'd got his news, as he forcibly expressed it, "off his chest," as if it were a nightmare or the indigestion; and as it was not his nature to bewail at length a fact, when it was a fact, he again referred to his little girl.

"Elsie ought to have been in now, oughtn't she?"

Mrs. Inman had really been getting a bit anxious herself as the evening was closing in, but she put a bold face on it, and said:

"She'll be safe enough. Maybe they're looking in at some shop-window or other, if the truth was known. They're sharp children, those Millwoods. Besides, what could have happened to them?"

"Happened?" said George, beginning to fret over this new cause—"a dozen things. Children wander further than they know, and get lost; go and fall down public-house cellars, get knocked down by cabs, get into the Park water, and—"

"Don't frighten me like that, George, for goodness' sake. Surely nothing's happened to her. Perhaps you'd better light your pipe, and go out and see if you can see her."

George agreed. Not that he really thought anything was wrong. "There's no accounting for children," was a frequent saying of his; "they do things that would frighten or kill grown-ups, and yet turn up all right in the end. Look at the smashes and scrapes I got into at school, and I'm not much the worse for them." He wanted a smoke, too, to soothe him. So he walked off down Bryanston Square and round it once or twice, then down George Street into Edgware Road. A little way along that he saw a small crowd dispersing at a street-corner, and a cab driving rapidly away. A policeman was conspicuous, just shutting up his note-book, but he was not communicative except so far as was necessary to make the people move away. So George addressed one of the crowd with—

"What's up, mate?"

"Little gal run over," was the reply. "Keb was jes' turning that corner, and she crossin' and run back. A decentish sort of woman picked her up, and the bobby put 'em both in the keb, and sent off to St. 'Lizabeth's Orspital."

"What was she like?"

"Oh! I dunno. Didn't see her close. Looked almost a baby, though, I heerd some one say."

George made more inquiries, but none seemed to know anything definite. The policeman vouchsafed that the little thing was senseless when he put her in the cab, and he didn't know her name and address. He had "the cabman's number, though." So he concluded to return home. When he got there he was surprised to find Tilda in great distress, weeping bitterly, and heard one of the little Millwood girls saying:

"Yes, Mrs. Inman, and the p'liceman picked her up and put her in a cab along with some old lady. And we ran home as fast as we could to tell you."

"What!" said George, jumping to conclusions. "It was our Elsie that was run over. Put on your bonnet and come along to St. Elizabeth's."

"You've heard of it then," sobbed Mrs. Inman. "What shall I do? Poor little dear! and all my fault letting her go out. What shall I do, to lose her like this?"

"Come," said George, encouragingly, "cheer up a bit. You haven't lost her yet. Put on your bonnet. We'll soon find out how it is." He felt heavy at heart himself, but he thought that giving the poor mother something to do was the best way to assuage the violence of her grief.

(*To be continued.*)

